# Supplementary material for: Sodium Benzoate Induces Fat Accumulation and Reduces Lifespan via the SKN-1/Nrf2 Signaling Pathway: Evidence from the Caenorhabditis elegans Model
Source: Nutrients. 2024 Oct 31;16(21):3753. doi: 10.3390/nu16213753 (PMC11547805; doi:10.3390/nu16213753)
Supplement: Supplementary file 1 [file nutrients-16-03753-s001.zip › nutrients-3237825-supplementary.pdf]

Supplementary Materials

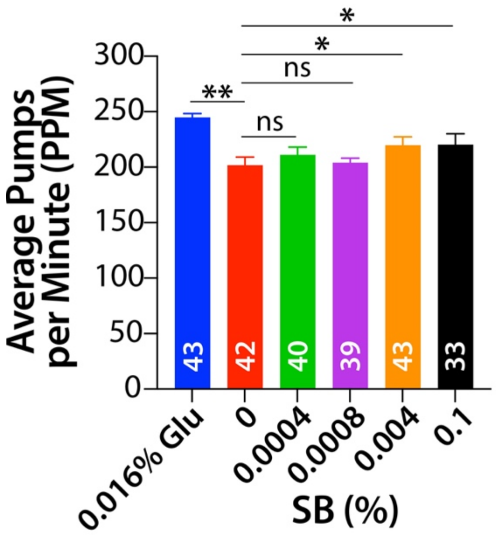

Figure S1. Average pharyngeal pumping rate of wild-type (N2) worms after 72 hours of sodium benzoate treatment. Y-axis represents the average pharyngeal pumping rate in pumps per minute. Error bars represent the standard error of 4 biological replicates. Not significant (ns), p > 0.05; *, 0.01 < p ≤ 0.05, **, 0.001 < p ≤ 0.01; ***, 0.0001 < p ≤ 0.001; **** p ≤ 0.0001.

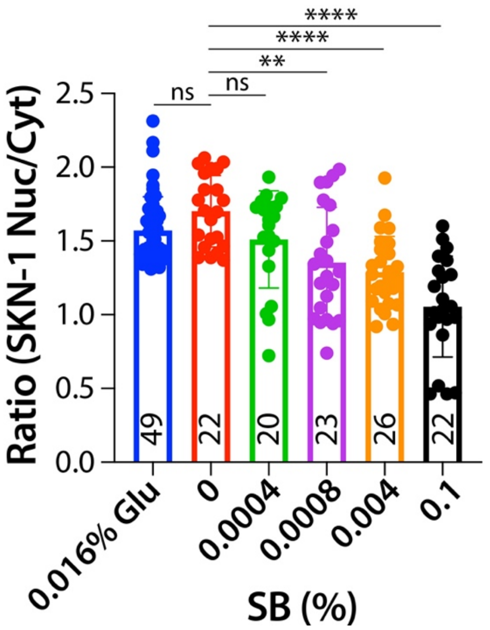

Figure S2. SB inhibits SKN-1 nuclear localization under normal conditions. The ratio of GFP intensities in the nucleus to the cytoplasm. Not significant (ns), p > 0.05; *, 0.01 < p ≤ 0.05, **, 0.001 < p ≤ 0.01; ***, 0.0001 < p ≤ 0.001; **** p ≤ 0.0001.
